# Potential Hypoglycemic and Antilipidemic Activity of Polyphenols from *Passiflora ligularis* (Granadilla)

**DOI:** 10.3390/molecules28083551

**Published:** 2023-04-18

**Authors:** Jaime Angel-Isaza, Juan Carlos Carmona-Hernandez, Clara Helena González-Correa, William Vicente Narváez-Solarte

**Affiliations:** 1 Research Group on Nutrition, Metabolism and Food Security (NUTRIMESA), Universidad de Caldas, Manizales 170001, Colombia; jaime.angel92@gmail.com (J.A.-I.); wnarvaez@ucaldas.edu.co (W.V.N.-S.); 2 Medical Research Group, Metabolism-Nutrition-Polyphenols (MeNutrO), Universidad de Manizales, Manizales 170004, Colombia; jucaca@umanizales.edu.co

**Keywords:** *Passiflora ligularis* (granadilla), *Camellia sinensis* (green tea), glucose, lipids, murine model

## Abstract

The consumption of fruits or by-products from plants of the Passifloraceae family has been associated with multiple health and nutritional benefits, due to their phenolic compound content. Likewise, the effects of polyphenols from *Camellia sinensis* (green tea) have been explored and are considered a reference for different biological actions of these bioactive substances. This study compared the hypoglycemic and antilipemic activity of polyphenol-rich extracts of *Passiflora ligularis* Juss (passion fruit) and *Camellia sinensis* (green tea) given to a group of Wistar rats induced to be overweight. The individuals were subjected to three doses of supplementation of both sources of polyphenols in the drinking water. An additional group without polyphenol supplementation served as a control group. Water consumption, weight gain, glycemia, cholesterol, serum triglycerides and percentage of fecal ethereal extracts were analyzed. Although *Passiflora ligularis* Juss had five times less polyphenol content than *Camellia sinensis*, rats fed doses of 2.5 and 3.0 g/L *Passiflora ligularis* Juss showed reduced glycemia by 16%, suggesting an antiglycemic activity similar to that of *Camellia sinensis*. On the other hand, higher doses of polyphenols from *Passiflora ligularis* Juss and *Camellia sinensis* significantly reduced triglyceride levels (*p* = 0.05) by more than 17% compared to the unsupplemented control group. The polyphenol-rich extracts produced effective inhibitory activity of lipemic metabolites with a reduction in the percentage of fecal lipids (*p* < 0.05), with no side effects on liver tissue. The 3.0 g/L dose produced the best result on signs of metabolic syndrome associated with excess weight. Polyphenols extracted from fresh Colombian passion fruit showed the potential to decrease metabolic syndrome risk factors in a murine model.

## 1. Introduction

Some of the most studied polyphenols are those found in the leaves of *Camellia sinensis* (green tea) due to a positive correlation between consumption and the low risk of metabolic disorders related to the cardiovascular system [1]. The most important polyphenols contained in *Camellia sinensis* are catechins, which include epigallocatechin (EGC), epicatechin gallate (ECG), epicatechin (EC), and epigallocatechin gallate (EGCG). The latter is the most relevant because it represents more than 40% of the catechins in the leaves of this plant. Polyphenols from this plant can reduce the accumulation of fat and lower negative effects from free radicals [2]. Polyphenols from tropical and Amazon plants could exhibit similar effects and provide other health benefits [3,4].

Green tea polyphenols, especially catechins, have been reported to be inhibitors of high blood pressure and high blood lipids in metabolic syndrome studies [5,6,7]. Although the precise mechanism is not established, the proposed inhibiting action is linked to lower intestinal cholesterol absorption and sluggish biosynthesis, as well as increased fecal excretion [6]. Comparative studies in search of other natural sources of beneficial polyphenols are needed.

The genus Passiflora includes nearly 500 species, some of which are of important economic and medicinal value. This fruit variety is cultivated in tropical and subtropical regions, especially in South America, the Caribbean, southern Florida, South Africa, and Asia [8]. Passiflora fruits have been used in popular medicine. Some of their main bioactive components are polyphenols, triterpenes, carotenoids, cyanogenic glycosides, polysaccharides, amino acids, essential oils, and different microelements [8]. Previous research on alcoholic extracts rich in polyphenols from *Passiflora ligularis* Juss containing ellagic acid, gallic acid, rutin, kaempferol, and caffeic acid showed powerful antioxidant, antidiabetic, and antimicrobial properties [9].

**Figure 1:**
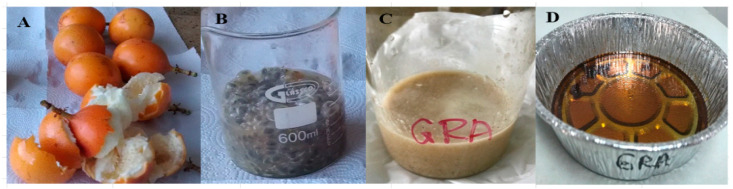
Colombian *Passiflora ligularis* (granadilla) treatment for polyphenol extraction. (**A**): fresh granadilla, (**B**): pulp and seeds, (**C**): homogenized pulp and seeds, (**D**): granadilla dry polyphenol extract.

Flavonoids, anthocyanins, phenolic acids, and tannins are found in Passiflora family fruits, such as *Passiflora edulis* Flavicarpa (maracuyá) and *Passiflora ligularis* Juss (granadilla) [10]. In Colombia, the cultivation of *Passiflora ligularis* Juss (granadilla) (Figure 1) is of economic importance, producing approximately 49,353 tons/year, with an average yield of 9.3 tons per ha/year [11].

*Passiflora ligularis* Juss is a potential source of polyphenols to be used for the benefit of animal and human health [10,12]. Some Passiflora, like granadilla and maracuyá, have been evaluated for their antioxidant and anti-inflammatory properties [3,4,13,14]. Previous research reported multiple biological actions of polyphenols, and their antioxidant effect is of important relevance [15]. Polyphenols act as direct or indirect antioxidants protecting the cell against oxidative stress. Another important action displayed by polyphenols is the anti-inflammatory effect. They inhibit specific reactions in the arachidonic cascade, blocking the production of eicosanoids that are active in physiological inflammatory events [15]. Polyphenols from edible sources (pulp, seeds, and edible flowers) are a quick and easy aid in dietetic or therapeutic processes: the inclusion of fresh tropical fruits in daily diets could help to prevent or reduce clinical different manifestations. The objective of the present work was to evaluate the total phenolic content and polyphenol-rich extract effect of *Passiflora ligularis* Juss (granadilla) on the glycemic and lipemic metabolism in a murine model in comparison with similar actions from polyphenols in *Camellia sinensis* (green tea).

## 2. Results

### 2.1. Total Polyphenol Content (TPC)

**Figure 2:**
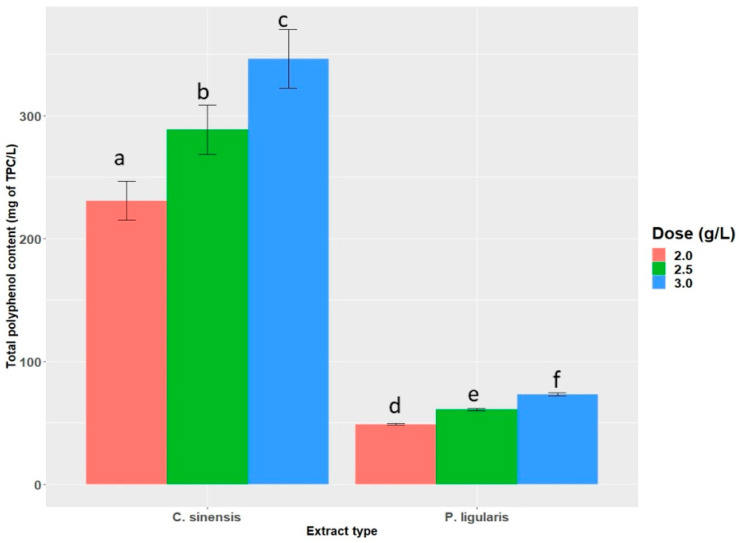
Total polyphenol content from both sources in each dose (mg of TPC/L of solution). Data are means (±standard deviations). Columns with different lowercase letters show statistically significant differences between them by the Duncan test (*p* < 0.05).

TPC in *Passiflora ligularis* Juss (dry extract) and *Camellia sinensis* (dry powder) was calculated as 24.43 (±0.39) and 115.42 (±8.28) mg GAE/g DM, respectively. Each drinkable dose for supplementation contained increasing amounts of total polyphenol content (Figure 2): 2.0, 2.5, and 3.0 g/L. Chromatographic analysis (UHPLC-MS) from previous studies by this research group highlighted the main phenolic compounds active in this Colombian Passiflora [4]. Based on UHPLC-MS values, 16 different phenolic compounds were detected in granadilla extracts. The highest concentrations corresponded to ferulic acid, epigallocatechin, epigallocatechin gallate, and (−)-epicatechin [4].

### 2.2. Water Consumption

**Figure 3:**
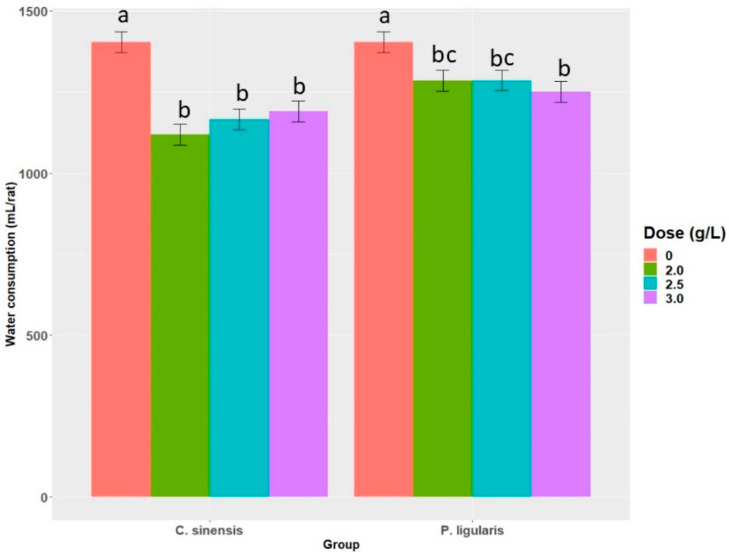
Total water consumption during the seven-week experimental period at different doses of supplementation with polyphenol-rich extracts. Data are means of volume (mL) consumed by animals in each group (*n* = 4) ± standard deviations. Columns with different lowercase letters show statistically significant differences between them by the Duncan test (*p* < 0.05).

The animals that consumed *Camellia sinensis* extract drank 18% less water. The *Passiflora ligularis* Juss group had 9.3% lower consumption compared to the group without supplementation and 9.1% higher consumption than the *Camellia sinensis* group. No difference was observed between the means of treatments. Figure 3 shows water consumption with the two polyphenol extracts in drinking water. Columns represent the total water volume (mL) consumed by each study group during the experimental period of a total of seven weeks.

### 2.3. Serum Glucose

There was an effect (*p* = 0.04) of the addition of polyphenol-rich extract of *Passiflora ligularis* Juss versus the control, showing a reduction in serum glucose of 21% and 30% in animals with *Passiflora ligularis* Juss extract when compared to the rats that received *C. sinensis* and the control group (Table 1). When comparing treatment doses within each extract, it was observed that the animals receiving 2.5 g/L and 3.0 g/L of the extract rich in polyphenols of *Passiflora ligularis* presented lower mean serum glucose in comparison with the control (*p* = 0.04).

### 2.4. Triglycerides

There was an effect (*p* = 0.01) of the addition of the extract, regardless of the source, versus the control, where the groups treated with *Camellia sinensis* and *Passiflora ligularis* Juss presented a reduction in mean triglyceride serum levels of 9% and 10%, respectively, compared to the control. Comparing the effect of extract dose, differences were found (*p* = 0.01), as displayed in Table 1. These showed a reduction in triglyceride levels in animals supplemented with 3.0 g/L of either extract compared to the lower dose of extract inclusion and control.

### 2.5. Cholesterol

No interaction was observed (Table 1) between the polyphenol-rich extract factors and the dose within each treatment (*p* = 0.32). There was no effect from the addition of polyphenol-rich extract of *Passiflora ligularis* Juss versus the control and treatments with *Camellia sinensis* (*p* = 0.77). In addition, no statistically significant differences were found between the dose within each extract (*p* = 0.69) for this variable.

### 2.6. Lipids in Feces

No interaction was observed between the polyphenol-rich extract factors versus the dose within each treatment (*p* = 0.39). There was an effect (*p* = 0.00) of the addition of the polyphenol extracts, regardless of the source, versus the control, where treatments with both extracts showed an increase of 5% in lipid content. Treatment with *Passiflora ligularis* Juss yielded the highest fecal lipid percentage accumulation in this study. All doses for both extracts were statistically higher than the control group, without differences between the dose of each extract (*p* = 0.00) as shown in Table 2.

### 2.7. Liver Histology

All the liver samples showed, after treatment at all concentrations, normal coloration, consistency, and homogeneity between lobes. They presented normal parenchyma without evidence of involvement in the centrilobular or periportal areas, and formed small, isolated foci of lymphocytes without necrosis or phagocytic cell accumulation.

**Figure 4:**
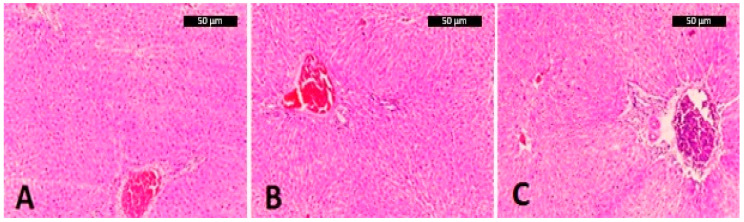
Effect of *Camellia sinensis* and *Passiflora ligularis* Juss (green tea and granadilla) polyphenol-extracts on hepatic histological condition after treatments. (**A**): group control, (**B**): 3.0 g/L green tea, (**C**): 3.0 g/L granadilla.

None of the histological sections exposed to different polyphenol doses from granadilla and green tea showed infiltration of fat drops, fibrosis, or necrosis, common characteristics of hepatic steatosis (Figure 4A–C represent treatments at the highest dose).

## 3. Discussion

Multiple studies have reported positive biological activity of polyphenols extracted from Passiflora [2,3,4,5,9,10,12,14,15]. Folin–Ciocâlteu methods are commonly used for the quantitation of total polyphenol content, and previous optimization studies had higher confidence in this technique [16,17]. Polar components extracted in alcohol solvents amino acid content in whey-based polyphenol extracts showed no interference with total polyphenol values tested by the Folin–Ciocâlteu method [18]. Previous UHPLC-MS studies done by this research team using in vitro and in vivo assays reported that the most abundant polyphenol compounds, of a total of 16 chromatographically identified compounds in passion fruit, were phenolic acids, xanthines, catechins, anthocyanins, (+)-catechin, (−)-epicatechin, ferulic acid, cyanidin 3-rutinoside, and quercetin 3-glucoside. The most abundant polyphenol in *Passiflora ligularis* was ferulic acid [3,4]. The present work focused on testing total polyphenol content in this Passiflora with possible inhibitory activity on metabolic syndrome biomarkers.

The highest polyphenol extract dose significantly reduced triglyceride concentration from an initial control of 119.0 mg/dL to 107.4 mg/dL in treatment with *Camellia sinensis* and to 107.8 mg/dL in the *Passiflora ligularis* Juss group. These results are consistent with Nakamura and Tonogai, who showed that supplementation with 1 g/kg polyphenols from grape seeds decreased triglyceride levels in rats from 80.9 mg/dL to 37.6 mg/dL after 28 days of treatment [19]. Anthocyanins and ellagic acid were the main polyphenols displaying pancreatic lipase maximum inhibitory action (IC50) of 111 and 108 mg/mL) and statistically reducing plasma triglycerides levels from 129 mg/dL, in the control group, to 72 mg/dL four hours after oral polyphenol supplementation. Mineo also observed lesser postprandial triglycerides concentration in rats supplemented with polyphenol-rich extracts from a hybrid berry of the genus *Rubus* (boysenberry) [20]. Serum triglycerides were statistically lowered in the group given doses starting at 0.05 g/kg of the polyphenol extract, without a specific report of characteristic polyphenols in the extracts.

Yokozawa, fed Wistar rats a high-cholesterol diet and increasing doses of green tea polyphenols for 5 weeks, and observed that free cholesterol and low-density lipoprotein (LDL) cholesterol were reduced, in addition to an increase in high-density lipoprotein (HDL) cholesterol with the administration of 2.5% polyphenols [21]. This decrease was also observed by Raederstorff with inclusion of 1% green tea EGCG. In the present study, only total cholesterol was measured and the percentage of lipids in the diet was slightly higher than that recommended in American Institute of Nutrition maintenance diet 93 (AIN-93M) for rats [22].

The beneficial effect of polyphenols on blood lipids has been associated with their ability to reduce lipid absorption [23,24]. A reduction in blood lipid was observed where the consumption of 2.0 g/L doses sufficed to increase 5% higher lipid excretion. Our results agree with Bose and Chen [24,25]. Similar results were observed with supplementation with 0.32% EGCG, which increased fecal lipids in mice by 20.4% [26]. Phenolic compounds in *Passiflora ligularis* Juss extracts exhibited a similar action in our results. Studies from Son showed increased cholesterol and triglyceride excretion related to the results of percentage lipid elimination in the present study [27]. Additionally, Ibitoye and Ajiboye reported low serum total cholesterol, triglycerides, and LDL in rats fed with a high-fructose diet and polyphenol supplementation [28].

A higher dose of *Passiflora ligularis* Juss extract significantly decreased blood glucose levels (*p* < 0.05). The *Camellia sinensis* polyphenol-supplemented group showed no significant differences, although blood glucose decreased 16% in the 3.0 g/L dose. In agreement, using Sprague Dawley rats that were given 1.1 g/L green tea catechins for 56 days, Ahmad observed decreased blood glucose in normal animals with rich-sucrose diets [29]. Similarly, Bose et al. (2008) fed a high-fat diet supplemented with EGCG to experimental mice and observed a reduction in glycemia. They concluded that polyphenols have the ability to counteract the negative effect on glycemia that could result from high lipid intake [24]. Other studies reported glucose concentration reduction of 62.09% and 15.00%, respectively, in rats fed high-fructose and ferulic acid diets for 13 weeks, also observing a reduction in the insulin resistance index [28,30].

Ahmad also proposes that glucose reduction, while consuming polyphenols, is due to the inhibition of the intestinal transporter of sodium-dependent glucose SGLT1 [29]. Researchers in another study found that polyphenols decrease the expression of gluconeogenic enzymes, such as phosphoenolpyruvate carboxykinase and glucose-6-phosphatase [31]. The control of oxidative stress by polyphenols may also be another factor that contributes to improve the utilization of glucose, since free radicals alter the signaling pathways of insulin by decreasing glucose uptake [32,33].

Plasma glycemic concentrations are positively regulated based on different studies [34,35,36]. For the specific effect of polyphenols from *Camellia sinensis* from tea, it has been reported as hypoglycemic activity based on concentrations ranging from 100 to 250 mg per kilogram of body weight in murine models [35,36]. Decreasing trends with respect to glucose concentrations vary depending on multiple factors. Dose-related studies show that there is still work to do in order to decipher the specific activity of polyphenols on main metabolic pathways [34]. Results in this work focused on different polyphenol concentrations (doses of 1.0 to 3.0 g/L) in drinking water rather than milligrams of polyphenol extracts per kilogram of body weight in rats.

Paquette states that polyphenol consumption increases insulin sensitivity. It prevents insulin elevation in the early phase, regulating an increase in the overall secretion [37]. This effect is important, because in the early stages of insulin resistance, plasma glucose levels remain normal thanks to a compensatory increase in the secretion of the hormone. Manzano proved that polyphenols reduce glucose and postprandial insulin and improve the capacity of peripheral tissues to respond to glucose uptake by insulin stimulation. They conclude that it is possibly due to the improvement in the translocation of the glucose transporter type 4 (GLUT4) from the low-density microsomes in the plasma membrane [38].

Alterations in the hepatic parenchyma such as necrosis or abnormal infiltration of macrophages were not observed. In addition, there was no evidence of fat droplets or fibrosis as indicative of steatosis. These results differ from those observed by Tan, Chen, and Bose who—using rats and mice fed high-fat diets—found signs of fatty liver disease [23,24,25]. The animals that were also supplemented with polyphenols had a lower incidence of hepatic steatosis. Dietary lipids were higher than those of the present experiment. The excess weight induction in this trial was probably not enough to produce lipid infiltration in the hepatocytes, as shown in the normal liver histology of the control group. With respect to a human equivalence dose for a 60 kg human, the 3.0 g/L of total polyphenols from fresh pulp and seeds of Colombian *Passiflora ligularis* corresponds to the content found in two fresh units of granadilla, as previously reported by Carmona-Hernandez et al. [3]. One factor to consider in the present study is that according to the equivalence for human doses, the polyphenols supplied to the animals were extracted from homogenized fresh passion fruit pulp and seeds, but in human consumption [3], the total phenolic content could be lower even with two units of the fruit, since some of the polyphenols will remain encapsulated in the fresh seeds and will not be digested.

## 4. Materials and Methods

The bioassay was conducted in the Bioterium of the Universidad de Caldas, in Manizales (Colombia) at 2100 m above sea level. The environmental conditions were between 18 °C and 25 °C with 50% relative humidity. Animals were born and bred in the Animal Care Department, and had 12 h of daylight. All procedures were approved by the Ethics Committee for Experimentation with Animals of the Faculty of Agricultural Sciences (approved and signed on 20 February 2018) from Universidad de Caldas.

### 4.1. Animals

The present study was done with 28 male Wistar rats, 120 days old, and an average initial weight of 288 ± 25 g. The sample size was determined aiming for a minimum statistical power of 80% (*p* < 0.05) [39,40]. The suggested size ranges were 27 to 36 animals [41]. A sample of 28 rats was chosen and animals were distributed into seven groups (*n* = 4). They were housed in groups of four in 800 cm^2^ plastic boxes covered with 5 cm-thick rice husk. Daily visits, records, and measurements were properly taken and registered for future data and statistical analysis.

### 4.2. Polyphenol Extraction and Total Polyphenol Content (TPC)

Granadilla pulp and seeds were homogenized (Oster^®^ Xpert Serier™, Milwaukee, WI, USA) and the mixture was placed in amber bottles and kept at 4 °C [42]. The homogeneous mixture was placed in a 70% acetone (Sigma Aldrich, St. Louis, MO, USA) solution in equivalent volumes, stirred for 20 min at 500 rpm (Dragon Lab MS-H Pro^®^, Beijing, China), sonicated for 15 min (Branson^®^ series MH™ model 3800, Danbury, CT, USA), and centrifuged at 3500 rpm for 15 min (Hermle^®^ Z 206A, Gosheim, Germany). The solvent was rota-evaporated (Scilogex^®^ RE 100 pro, Rocky Hill, CT, USA) and the resulting polyphenol-rich extract was dried at 50 °C for 96 h (Inducell^®^ LSIS-S, Grafelfing, Germany). The green tea polyphenol extract, from dry, nonfermented leaves of *Camellia sinensis*, was purchased from a commercial company. The 95% total polyphenol extract contained 75% catechins with 45% EGCG as its major component (Oleo Especias, Jalisco, Mexico). The product was kept out of direct light and in a well-ventilated container.

TPC was quantified based on the Folin–Ciocâlteu (F-C) (Pan Reac Appli Chem, ITW reagents, Darmstadt, Germany) procedures [17]. Granadilla and green tea samples of 1 mL of each solubilized extract were added to 1 mL of the F-C reagent (10%), vortexed for 15 s, and mixed with 2 mL of sodium carbonate (3.5%) (Sigma Aldrich, St. Louis, MO, USA). The reaction lasted 90 min in the dark. All assays were done in triplicate. Gallic acid (Sigma Aldrich, St. Louis, MO, USA) was used as a comparable standard based on a calibration curve. Results are reported as milligrams of gallic acid equivalents per gram of dry material (mg GAE/g DM ± standard deviation). The samples were read on a UV/vis spectrophotometer at 655 nm (Optizen POP^®^, Daejeon, Republic of Korea).

Once the phenolic content from both sources (granadilla and green tea) was determined, dosage of drinkable polyphenol supplementation was calculated based on a solid-liquid dilution. Dry granadilla extracts and green tea samples were weighed and dissolved in water, homogenized, and kept at 4 °C until delivery to the study groups. Polyphenols in each dose for supplementation are reported as milligrams of total polyphenol content per liter of solution (mg TPC/L).

### 4.3. Diets

The study lasted 42 days. The animals received commercial food and water ad libitum [43]. Adaptation and excess weight induction was achieved at day 14. Water sucrose content provided was 30%, pursuing an 18% weight gain [44]. The rats were weighed and fed to reach a homogenized average weight of 350 ± 2 g. Treatments were distributed considering an experimental unit, with a 2 × 3 + 1 factorial model consisting of two sources of polyphenol extract (*Passiflora ligularis* and *Camellia sinensis*), three extract doses (2.0, 2.5, and 3.0 g/L) and one control group. Every week, the animals were weighed and water consumption measured.

At day 42, samples of feces were collected and packed in plastic bags for 4 h, then dried in an oven (Herathem Thermo^®^, Langenselbold, Germany) at 55 °C for 24 h. Each sample was weighed (1.5 g portions) and wrapped in filter paper to perform total fat determination by the Soxhlet method [45]. The animals were euthanized following the recommendations for the care and use of laboratory animals of the National Research Council [46]. The anesthetic process was done with CO_2_ at a concentration of 70% for one minute. Blood from each experimental unit was collected in tubes with glycolytic inhibitors (5 mg of sodium fluoride and 4 mg of potassium oxalate). The serum was obtained by centrifugation (Hettich^®^ EBA 200, Tuttlingen, Germany) at 4500 rpm for 5 min. Glucose, cholesterol and triglycerides were quantified in a semiautomatic clinical chemistry analyzer (Biosystems^®^ BTS-350, Costa Brava, Barcelona, Spain), using each of the specific enzyme kits.

Liver tissue samples were taken to evaluate the degree of steatosis. They were preserved in 10% buffered formalin, then dehydrated in ethyl alcohol at 70%, 80%, 90% and 100% and diaphanid in two xylol baths for inclusion in paraffin. Rotary microtome slides of 4 μm thickness were obtained to be stained with hematoxylin and eosin. The histological plates were read with an optical microscope (Leica^®^ MD200, Wetzlar, Germany). The degree of hepatic steatosis was determined according to the methodology described by Brenes-Pino [47].

### 4.4. Statistical Analysis

Results of quantitative variables were analyzed through analysis of variance. In variables that showed significant statistical difference, the means were compared using the Duncan test. Means of the variables that did not show statistical normality were tested with Shapiro–Wilk tests (*p* < 0.05). All statistical procedures were performed using the licensed statistical program STATA 12^®^. The graphs were made using the ggplot2 package of the R studio program.

## 5. Conclusions

Although total polyphenol content in the extracts from granadilla was fivefold lower than the polyphenol content in green tea, the polyphenol extract of *Passiflora ligularis* Juss (granadilla) had in vivo activity similar to the activity of polyphenols from *Camellia sinensis* (green tea). Granadilla polyphenols decreased risk factors of metabolic syndrome in Wistar rats, favoring lower concentrations of lipids and glucose in blood, at the same time preventing the absorption of fat at the intestinal level. It was observed that the dose of extract rich in polyphenols (3.0 g/L), regardless of its origin, showed better action on the signs of the metabolic syndrome associated with excess weight without causing deleterious effects in liver tissue. Future research approaches should include validation of the polyphenol content in fresh fruit or as supplemented products in human studies. An aggregate value of this research direction is that the consumption of fresh fruit, besides the polyphenol content, provides other beneficial nutrients, such as vitamins, minerals, fiber and water.

## Figures and Tables

**Table 1:** Effect of supplementation with polyphenol-rich extracts on metabolic components.

Group	Dose (mL/L)	Cholesterol	Triglycerides	Glucose
		(mg/dL)	(mg/dL)	(mg/dL)
Control	0.0	106.5 ± 9.6	119.0 ± 3.7 ^b^	187.0 ± 42.8 ^b^
*Passiflora ligularis*	2.0	111.5 ± 6.4	112.5 ± 16.0 ^b^	144.7 ± 23.2 ^ab^
	2.5	110.5 ± 14.7	111.5 ± 12.4 ^b^	123.2 ± 18.6 ^a^
	3.0	98.3 ± 10.8	98.3 ± 57 ^a^	122.2 ± 33.3 ^a^
*Camellia sinensis*	2.0	108.5 ± 13.7	115.2 ± 13.8 ^b^	163.7 ± 21.7 ^b^
	2.5	109.7 ± 14.1	109.7 ± 6.1 ^b^	165.5 ± 32.3 ^b^
	3.0	112.2 ± 11.6	98.5 ± 3.1 ^a^	156.7 ± 17.7 ^b^

Cholesterol, triglycerides, and glucose blood levels in Wistar rats. Data are means (±standard deviations). Means of treatments within the same column followed by different lowercase letters are statistically different from each other by Duncan’s test (*p* < 0.05).

**Table 2:** Effect on feces lipid percentages after supplementation with polyphenol extracts at different doses.

Group	Dose (mL/L)	Lipids (%)
Control	0.0	2.45 ± 0.02 ^a^
*Passiflora ligularis*	2.0	2.56 ± 0.03 ^b^
	2.5	2.57 ± 0.04 ^b^
	3.0	2.57 ± 0.03 ^b^
*Camellia sinensis*	2.0	2.55 ± 0.01 ^b^
	2.5	2.55 ± 0.01 ^b^
	3.0	2.56 ± 0.03 ^b^

Means of treatments within the same column followed by different lowercase letters are statistically different from each other by Duncan’s test (*p* < 0.05).

## Data Availability

Data will be shared upon request.

## Sample Availability

Samples of the compounds are not available from the authors.
